# Determination of ADAMTS13 and Its Clinical Significance for ADAMTS13 Supplementation Therapy to Improve the Survival of Patients with Decompensated Liver Cirrhosis

**DOI:** 10.4061/2011/759047

**Published:** 2011-07-18

**Authors:** Masahito Uemura, Yoshihiro Fujimura, Saiho Ko, Masanori Matsumoto, Yoshiyuki Nakajima, Hiroshi Fukui

**Affiliations:** ^1^Third Department of Internal Medicine, Nara Medical University, 840 Shijo-cho, Kashihara, Nara 634-8522, Japan; ^2^Department of Blood Transfusion Medicine, Nara Medical University, Kashihara, Nara 634-8522, Japan; ^3^Department of Surgery, Nara Medical University, Kashihara, Nara 634-8522, Japan

## Abstract

The liver plays a central role in hemostasis by synthesizing clotting factors, coagulation inhibitors, and fibrinolytic proteins. Liver cirrhosis (LC), therefore, impacts on both primary and secondary hemostatic mechanisms. ADAMTS13 is a metalloproteinase, produced exclusively in hepatic stellate cells, and specifically cleaves unusually large von Willebrand factor multimers (UL-VWFM). Deficiency of ADAMTS13 results in accumulation of UL-VWFM, which induces platelet clumping or thrombi under high shear stress, followed by sinusoidal microcirculatory disturbances and subsequent progression of liver injuries, eventually leading to multiorgan failure. The marked imbalance between decreased ADAMTS13 activity (ADAMTS13 : AC) and increased production of UL-VWFM indicating a high-risk state of platelet microthrombi formation was closely related to functional liver capacity, hepatic encephalopathy, hepatorenal syndrome, and intractable ascites in advanced LC. Some end-stage LC patients with extremely low ADAMTS13 : AC and its IgG inhibitor may reflect conditions similar to thrombotic thrombocytopenic purpura (TTP) or may reflect “subclinical TTP.” Hence, cirrhotic patients with severe to moderate deficiency of ADAMTS13 : AC may be candidates for FFP infusion as a source of ADAMTS13 or for recombinant ADAMTS13 supplementation. Such treatments may improve the survival of patients with decompensated LC.

## 1. Introduction

The liver is a major source of clotting and fibrinolytic proteins and plays a central role in thromboregulation [1–4]. Liver diseases, hence, impact on both primary and secondary hemostatic mechanisms. Because the hemostatic system is normally in a delicate balance between pro-hemostatic and antihemostatic processes, advanced liver cirrhosis (LC) patients experience multiple changes in the hemostatic system that may lead to either bleeding or thrombosis [1–4]. Despite clinical evidence of increasing bleeding tendency in LC patients, many facts indicate local and systemic hypercoagulability including portal or hepatic vein thrombosis, pulmonary embolism, and deep vein thrombosis, which are closely related to microcirculatory disturbances [4]. Deficiency of anticoagulant proteins and high levels of several procoagulant factors may favor hypercoagulability [4], but the mechanisms underlying this disorder have not been fully elucidated.

ADAMTS13 (**a** **d**isintegrin-like **a**nd **m**etalloproteinase with **t**hrombo**s**pondin type-1 motifs **13**) is a metalloproteinase that specifically cleaves multimeric von Willebrand factor (VWF) between Tyr1605 and Met1606 residues in the A2 domain [5, 6]. In the absence of ADAMTS13 activity (ADAMTS13 : AC), unusually large VWF multimers (UL-VWFMs) are released from vascular endothelial cells (ECs) and improperly cleaved, causing them to accumulate and to induce the formation of platelet thrombi in the microvasculature under conditions of high shear stress. Currently, a severe deficiency in ADAMTS13 : AC, which results either from genetic mutations in the *ADAMTS13* gene (Upshaw-Schulman syndrome, (USS)) [5–8] or acquired autoantibodies against ADAMTS13 [9, 10], is thought to be a specific feature of thrombotic thrombocytopenic purpura (TTP) [5–12].

In 2000, we demonstrated that a decreased plasma ADAMTS13 : AC in patients with cirrhotic biliary atresia can be fully restored after liver transplantation, indicating that the liver is the main organ producing ADAMTS13 [13]. One year later, northern blot analysis showed that the 4.6-kilobase ADAMTS13 mRNA was highly expressed in the liver [7, 14, 15], and subsequently both *in situ* hybridization and immunohistochemistry clearly indicated that ADAMTS13 is produced exclusively in hepatic stellate cells (HSCs) [16]. Platelets [17], vascular ECs [18], and kidney podocytes [19] have also been implicated as ADAMTS13-producing cells, but the amount produced by these cell types in the liver appears to be far less than that produced by HSC. 

Mannucci et al. [20] originally reported a reduction of the ADAMTS13 : AC in advanced LC. Since HSCs were shown to be the major producing cells in the liver [16], much attention has been paid to the potential role of ADAMTS13 in the pathophysiology of liver diseases associated with sinusoidal and/or systemic microcirculatory disturbance [21–35]. ADAMTS13 : AC significantly decreased in patients with hepatic veno-occlusive disease (VOD) [22, 23], alcoholic hepatitis [24–27], liver cirrhosis [29, 30], and those undergoing living-donor-related liver transplantation [31–33] and partial hepatectomy [34]. Furthermore, hepatitis C virus- (HCV-) related LC patients with ADAMTS13 inhibitor (ADAMTS13 : INH) typically developed TTP [35]. Once patients with LC develop a decompensated condition, the risk of early mortality sharply increases for specific life-threatening complications such as ascites, hepatic encephalopathy, sepsis, hepatorenal syndrome, or hepatopulmonary syndrome [36].

In this paper, we will focus on the importance of ADAMTS13 determination for a better understanding of pathophysiology and/or for possible therapeutic approaches of ADAMTS13 supplementation to improve survival in patients with advanced LC.

## 2. Hepatic Microcirculation and Hypercoagulable State in LC

Hepatic microcirculation compromises a unique system of capillaries, called sinusoids, which are lined by three different cell types: sinusoidal endothelial cells (SECs), HSC, and Kupffer cells [37]. The SEC modulates microcirculation between hepatocytes and the sinusoidal space through the sinusoidal endothelial fenestration. The SEC has tremendous endocytic capacity, including VWF and the extracellular matrix, and secretes many vasoactive substances [37]. The HSC is located in the space of Disse adjacent to the SEC and regulates sinusoidal blood flow by contraction or relaxation induced by vasoactive substances [38]. Kupffer cells are intrasinusoidally located tissue macrophages and secrete potent inflammatory mediators during the early phase of liver inflammation [37]. Intimate cell-to-cell interaction has been found between these sinusoidal cells and hepatocytes [37, 38]. In LC, a sinusoidal microcirculatory disturbance occurs when the normal hepatic structure is disrupted by fibrin deposition [39] or by impaired balance between the action of vasoconstrictors and vasodilators in hepatic vascular circulation [37]. Studies have shown that cirrhotic liver exhibits a hyperresponse to vasoconstrictors, including catecholamine, endothelin, and leukotrienes D_4_ [37].

Vascular endothelial cells play a pivotal role in hemostasis and thrombosis [5, 6]. VWF is a marker of endothelial cell activation (damage) and plays an essential role in hemostasis [5, 6]. In the normal state, VWF immunostaining is usually positive in large vessels but negative in the SEC [40]. On the occurrence of liver injury accompanied by a necroinflammatory process, the SEC becomes positive for VWF, presumably in association with the capillarization of hepatic sinusoids [39]. Subsequently, platelets adhere to subendothelial tissue mediated by UL-VWFM [5, 6]. ADAMTS13 then cleaves UL-VWFM into smaller VWF multimers [5, 6]. This interaction of ADAMTS13 and UL-VWFM is, indeed, the initial step in hemostasis [5, 6]. 

In patients with LC, circulating plasma VWF levels are extremely high [41, 42]. In liver tissue from cirrhotics [43] and even from the early stages of alcoholic liver diseases [44], VWF immunostaining shows positive cells predominantly at the scar-parenchyma interface, within the septum, and in the sinusoidal lining cells. Actually, portal or hepatic vein thrombosis is often observed in advanced LC routinely screened with Doppler ultrasound [45], and, in cirrhotic liver removed at transplantation, intimal fibrosis suggesting hepatic and portal vein thrombosis was frequently observed [46]. An autopsy series revealed microthrombi in one or multiple organs in one-half of cirrhotics [47]. Such a hypercoagulable state in liver diseases may be involved in hepatic parenchymal destruction, the acceleration of liver fibrosis and disease progression [4], leading to hepatorenal syndrome, portopulmonary hypertension, and spontaneous bacterial peritonitis [48]. 

Systemically, deficiency of anticoagulant proteins (antithrombin, protein C, and protein S) and the high levels of several procoagulant factors (factor VIII and VWF) may contribute to hypercoagulability in patients with LC [4]. Locally, the SEC dysfunction could lead to the development of a hypercoagulable state at the hepatic sinusoids corresponding to the site of liver injury, even in the face of a systemic hypocoagulable state [4]. Considering that ADAMTS13 is synthesized in HSC and its substrate, UL-VWFM, is produced in transformed SEC during liver injury, decreased plasma ADAMTS13 : AC may involve not only sinusoidal microcirculatory disturbances, but also subsequent progression of liver diseases, finally leading to multiorgan failure. Based on these findings, it is of particular interest to evaluate the activity of plasma ADAMTS13 : AC in LC patients.

## 3. Cleavage of UL-VWFM by ADAMTS13

**Figure 1:**
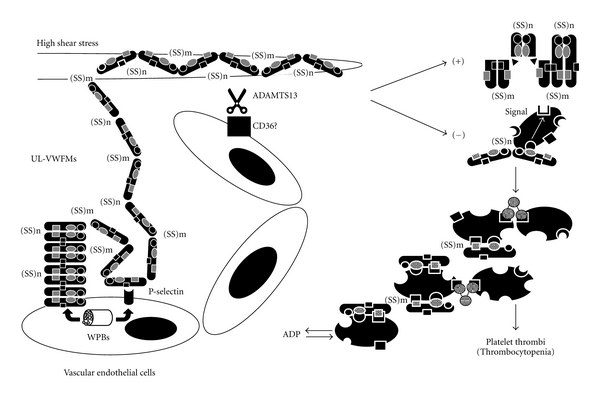
Proposed mechanism of platelet thrombi under high shear stress in the absence of ADAMTS13 : AC. Unusually large von Willebrand factor multimers (UL-VWFMs) are produced in vascular endothelial cells (ECs) and stored in Weidel-palade bodies (WPBs). UL-VWFMs are released from WPBs into the circulation upon stimulation by cytokines, hypoxia, DDAVP, and epinephrine. P-selectin that comigrates from WPBs anchors UL-VWFMs on the vascular EC surface. Under these circumstances, high shear stress changed the molecular conformation of UL-VWFMs from a globular to an extended form, allowing ADAMTS13 to access this molecule. In the absence of ADAMTS13 : AC, UL-VWFMs remain uncleaved, allowing them to excessively interact with platelet glycoprotein (GP)Ib*α* and activate platelets via intraplatelet signaling, which result in the formation of platelet thrombi. (Partially modified from Fujimura et al., [49]).

Although the mechanism by which TTP develops in the absence of ADAMTS13 : AC has not been fully elucidated, accumulating evidence has provided a hypothesis as illustrated in Figure 1 [49]. UL-VWFMs are produced exclusively in vascular ECs and stored in an intracellular organelle termed Weidel-palade bodies (WPBs) and then released into the circulation upon stimulation. Under physiological conditions, epinephrine acts as an endogenous stimulus, but under nonphysiological conditions, DDAVP (1-deamino-8-D-arginine vasopressin), hypoxia, and several cytokines such as interleukin IL-2, IL-6, IL-8, and tumor necrosis factor- (TNF-) *α* act as stimuli that upregulate VWF release. Once ECs are stimulated, UL-VWFMs and P-selectin, both stored in WPBs, move to the membrane surface of ECs, where P-selectin anchors UL-VWFMs on the ECs surface [50]. Under these circumstances, high shear stress generated in the microvasculature induces a change in the UL-VWFM from a globular to an extended form [51]. The ADAMTS13 protease efficiently cleaves the active extended form of UL-VWFM between the Tyr1605 and Met1606 residues in the A2 domain [52]. In this context, it has been postulated that multiple exocites within the disintegrin-like/TSP1/cysteine-rich/spacer (DTCS) domains of ADAMTS13 play an important role in interacting with the unfolded VWF-A2 domain [53]. ADAMTS13 may more efficiently cleave newly released UL-VWFMs that exist as solid-phase enzymes anchored to the vascular EC surface by binding to CD36, because CD36 is a receptor for TSP1, which is a repeated domain within the ADAMTS13 molecule [54]. When ADAMTS13 activity is reduced, UL-VWFM interacts more intensively with platelet GPIb and generates signals that further accelerate platelet activation [5, 6]. A series of these reactions leads to platelet microaggregates and thrombocytopenia. However, little information has been available on the cleavage of the UL-VWFMs by ADAMTS13 in the sinusoidal microcirculation in LC.

## 4. Assays for Plasma ADAMTS13 : AC and ADAMTS13 : INH

ADAMTS13 : AC was determined with a classic VWFM assay in the presence of 1.5 mol/L urea using purified plasma-derived VWF as a substrate according to the method described by Furlan et al. [55], and the detection limit of this assay was 3% of the normal control in our laboratory [56]. In 2005, we developed a novel chromogenic ADAMTS13-act-ELISA using both an N- and C-terminal tagged recombinant VWF substrate (termed GST-VWF73-His). This assay was highly sensitive, and the detection limit was 0.5% of the normal control [57]. Plasma ADAMTS13 : AC levels highly correlated between VWFM assay and ADAMTS13-act-ELISA (mean ± SD, 102 ± 23% versus 99.1 ± 21.5%, *r* ^2^ = 0.72, *P* < .01) [57]. No interference of the ADAMTS13-act-ELISA occurred even in the presence of hemoglobin, bilirubin, or chylomicrons in the samples, thus enabling distinction from the FRETS-VWF73 assay [58]. Because of its high sensitivity, easy handling, and lack of interference from plasma components, the ADAMTS13-act-ELISA would be recommended for routine laboratory use. 

The ADAMTS13 : INH has also been evaluated with the chromogenic act-ELISA by means of the Bethesda method [59]. Prior to the assay, the test samples were heat-treated at 56°C for 60 min to eliminate endogenous enzyme activity, mixed with an equal volume of intact normal pooled plasma, and incubated for 2 hours at 37°C. The residual enzyme activity is measured after incubation. One Bethesda unit is defined as the amount of inhibitor that reduces activity by 50% of the control value, and values greater than 0.5 U/mL are significant.

## 5. Thrombocytopenia, Determination of ADAMTS13 : AC, and Its Clinical Significance in LC

### 5.1. Thrombocytopenia

**Figure 2:**
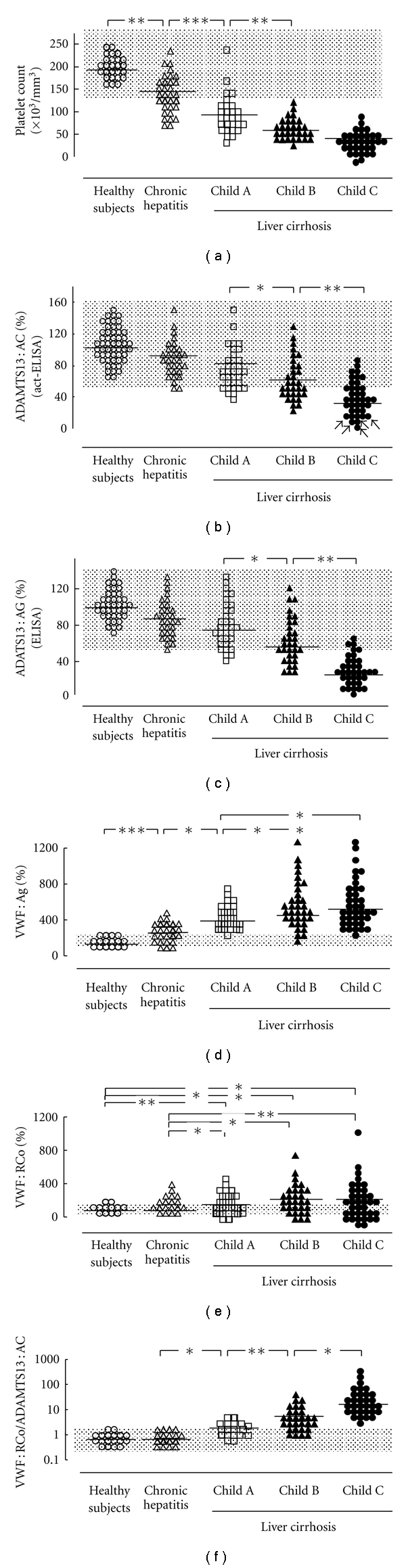
Platelet counts and plasma levels of ADAMTS13 : AC and its related parameters in patients with chronic liver diseases. Platelet counts decreased with the severity of chronic liver diseases, but no difference was found between Child B and C (a). Plasma ADAMTS13 : AC determined by ELISA progressively decreased with worsening cirrhosis (b). Arrows indicate patients whose plasma ADAMTS13 : AC was extremely low (< 3% of normal control by VWFM assay). The ADAMTS13 : AG levels determined by ELISA also decreased with increasing cirrhosis severity (c), which highly correlated with ADAMTS13 : AC measured by the act-ELISA (*r* = 0.715, *P* < .001). The VWF : Ag increased with the progression of chronic liver diseases, but the difference between Child B and C did not reach statistical significance (d). The VWF : RCo is higher in liver cirrhosis patients than that in patients with chronic hepatitis and healthy subjects, but it did not differ among subgroups within liver cirrhosis (e). The VWF : RCo relative to ADAMTS13 : AC progressively increased with worsening chronic liver disease (f). Open circles: normal controls; open triangles: chronic hepatitis; open squares: cirrhosis with Child A; closed triangles: cirrhosis with Child B; closed circles: cirrhosis with Child C. Shaded area shows normal range. ADAMTS13 : AC : ADAMTS13 activity, ADAMTS13 : AG = ADAMTS13 antigen. VWF : Ag = von Willebrand factor antigen, VWF : RCo = von Willebrand factor ristocetin cofactor activity; *P < .05, **P < .01, and ***P < .001 significantly different between the two groups. (Partially modified from Uemura et al., [30]).

It is well accepted that thrombocytopenia gradually progresses as functional liver capacity decreases [30, 60] (Figure 2(a)). The pathogenesis of thrombocytopenia in LC includes splenic sequestration in portal hypertension [61], impaired platelet production due to decreased synthesis of thrombopoietin in the liver [62] or due to myelosupression resulting from HCV infection [63], folic acid deficiency, or ethanol chronic consumption [64], which has a negative effect on megacaryocytopoiesis. However, our recent studies have provided evidence that in patients with advanced LC, elevated plasma levels of UL-VWFM enhance high-shear stress-induced platelet aggregation, resulting in thrombocytopenia [30].

### 5.2. ADAMTS13 : AC

Our study showed that ADAMTS13 : AC decreased with increasing severity of cirrhosis [30] (Figure 2(b)). The values determined by act-ELISA correlated well with those of the classical VWFM assay and also closely correlated with ADAMTS13 antigen determined by the antigen-ELISA. These results confirmed that both ADAMTS13 activity and antigen decreased with increasing cirrhosis severity [30] (Figures 2(b) and 2(c)), which are consistent with findings described by Feys et al. [29]. In contrast, Lisman et al. showed that both ADAMTS13 activity and antigen levels were highly variable; however, they did not distinguish between patients with varying degrees of cirrhosis [28]. It is unclear why they reached different conclusions from ours. One possible explanation relates to different etiologies: a majority of our patients developed cirrhosis secondary to HCV infection, whereas in their study one-half of the patients suffered from alcohol abuse-related cirrhosis. Further, the techniques used to determine ADAMTS13 : AC differed between our study [55–57] and theirs [65]. It is assumed that the collagen binding assay they used can be highly influenced by the increased amount of VWF : Ag in tested cirrhotic plasmas [29], because the substrate in this assay is intact multimeric VWF. In this regard, our act-ELISA is performed using VWF73-based fusion protein, termed GST-VWF73-His, which is readily cleaved by ADAMTS13 without any protein denaturant, and therefore the increased amount of VWF : Ag in tested plasmas does not interfere with the assays [57].

**Figure 3:**
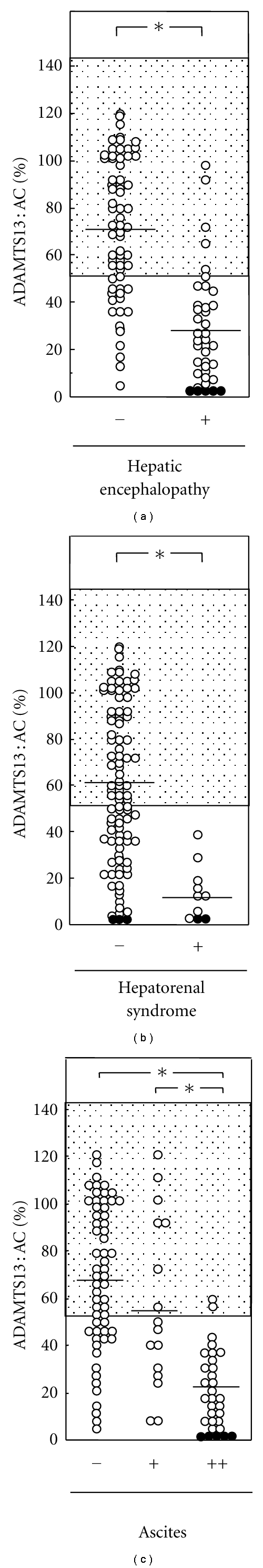
Relationship of ADAMTS13 : AC to the presence or absence of hepatic encephalopathy, hepatorenal syndrome, and ascites in patients with liver cirrhosis. The ADAMTS13 : AC was significantly lower in LC patients with hepatic encephalopathy (a) and hepatorenal syndrome (b) than that those without. Moreover, patients with refractory ascites had lower ADAMTS13 : AC than those without ascites or those with easily mobilized ascites (c). Closed circles indicate patients whose plasma ADAMTS13 : AC was extremely low (< 3% of normal control by VWFM assay). ADAMTS13 : AC: ADAMTS13 activity; *P < .001 significantly different between the two groups. (Partially modified from Uemura et al., [30]).

As shown in Figure 3, ADAMTS13 : ACs were significantly lower in LC patients with hepatic encephalopathy (Figure 3(a)), hepatorenal syndrome (Figure 3(b)), and severe esophageal varices than those without [30]. Moreover, patients with refractory ascites had lower ADAMTS13 : AC levels than patients without ascites or those with easily mobilized ascites (Figure 3(c)). A multivariate analysis using all significant baseline parameters determined by the univariate analysis, excluding the Child-Pugh score, showed spleen volume, blood ammonia, and serum creatinine independently correlated with ADAMTS13 : AC. As a second step, the three parameters that contribute to the Child-Pugh classification (total bilirubin, albumin, and prothrombin time) were replaced by the Child-Pugh score. As a result, the Child-Pugh score and spleen volume were independently selected, indicating that ADAMTS13 : AC is closely related to the severity of liver disease and splenomegaly in cirrhotic patients [30].

### 5.3. VWF : Ag and VWF Multimer Patterns

Plasma levels of VWF : Ag substantially increase as liver diseases progress (Figure 2(d)) [30], as previously reported [41, 42]. This is presumably attributed to sinusoidal and/or extrahepatic endothelial damage induced by endotoxin and cytokines [41, 42, 66, 67]. The VWF : RCo was higher (Figure 2(e)) [30], but the ratio of VWF : RCo/VWF : Ag was lower in LC patients than that in healthy subjects. These findings suggest that increased VWF : Ag appears less functional in LC patients [30], which are consistent with previous reports [28]. Nevertheless, our study has clearly shown that the ratio of VWF : RCo/ADAMTS13 : AC progressively increases with the worsening of chronic liver diseases (Figure 2(f)), further intensifying an enhanced thrombogenesis with the progression of liver dysfunction and thrombocytopenia [30]. 

 With regard to VWF multimers, the higher molecular weight multimer showed greater degradation than in healthy controls, thus maintaining normal enzyme-to-substrate (ADAMTS13/UL-VWFMs) ratio to maintain blood fluidity [29]. We showed that there were three different VWFM patterns in LC patients with lower ADAMTS13 : AC (<50 % of controls): normal-VWFM was detected in 53%, degraded-VWFM in 31%, and UL-VWFM in 16% (Table 1) [30]. UL-VWFM-positive patients showed the lowest ADAMTS13 : AC and the highest values of serum creatinine, blood urea nitrogen, and blood ammonia. In addition, LC patients with UL- and normal-VWFM had higher levels of VWF : RCo and Child-Pugh score and lower values of cholinesterase and hemoglobin than those with degraded-VWFM [30] (Table 1). The pattern, therefore, appears to shift from degraded- to normal-VWFM, and finally to UL-VWFM as functional liver capacity and renal function deteriorates, indicating that advanced LC may be a predisposing state toward platelet microthrombi formation, even in the absence of clinically overt thrombotic events [30].

## 6. Mechanism of Decreased ADAMTS13 : AC in LC Patients

The mechanism responsible for the decrease in ADAMTS13 : AC in advanced LC may include enhanced consumption due to the degradation of large quantities of VWF : AG [20], inflammatory cytokines [68, 69], and/or ADAMTS13 plasma inhibitor [9, 10]. It is controversial whether ADAMTS13 deficiency is caused by decreased production in the liver; Kume et al. reported that HSC apoptosis plays an essential role in decreased ADAMTS13 : AC using dimethylnitrosamine-treated rats, but not carbon tetrachloride- (CCl_4_-) treated animals [70], whereas Niiya et al. found upregulation of ADAMTS13 antigen and proteolytic activity in liver tissue using rats with CCl_4_-induced liver fibrosis [71]. We observed the inhibitor of ADAMTS13 in 83% of patients with severe to moderate ADAMTS13 deficiency, but its inhibitory activity was in a marginal zone between 0.5 and 1.0 BU/mL in most cases except in cases of a TTP patient (2.0 BU/mL) and a patient with severe ADAMTS13 deficiency (3.0 BU/mL) [30]. Interestingly, IgG-type autoantibodies specific to purified plasma derived-ADAMTS13 were detected by Western blotting only in five end-stage cirrhotics with severe ADAMTS13 deficiency (<3%) corresponding to TTP [30]. One patient showed an apparent TTP [35], while the other four cirrhotics did not show apparent clinical features of TTP but had complications of hepatorenal syndrome, spontaneous bacterial peritonitis (SBP), marked inflammation together with cytokinemia, and advanced hepatocellular carcinoma (HCC) [30]. Various clinical conditions, including infection, malignancies, and certain drugs, can lead to acquired TTP [72]. In advanced LC patients, endotoxemia is frequently detected [42, 73], and SBP sometimes occurs [74]. HCC is highly complicated as the cirrhotic stage progresses [75], suggesting a high-risk state of platelet microthrombi formation. Some end-stage LC patients with extremely low ADAMTS13 : AC and its IgG inhibitor may reflect conditions similar to TTP or may reflect “subclinical TTP” [21]. Further studies will be necessary to clarify whether inhibitors other than the IgG inhibitor might be involved in cirrhotics with lower ADAMTS13 : AC. 

Alternatively, cytokinemia [25, 68, 69, 76] and endotoxemia [25, 77] are additional potential candidates for decreasing plasma ADAMTS13 : AC. Recent investigations demonstrated that IL-6 inhibited the action of ADAMTS13 under flow conditions and both IL-8 and TNF-*α* stimulated the release of UL-VWFM in human umbilical vein endothelial cells *in vitro* [68]. It remains to be clarified whether IL-6 directly hampers the cleavage of UL-VWFM or downregulates gene expression of ADAMTS13 with modification of promoter activity. IFN-*γ*, IL-4, and TNF-*α* also inhibit ADAMTS13 synthesis and activity in rat primary HSC [69]. In addition, ADAMTS13 deficiency associated with inflammation promoted formation of UL-VWFM [78], and intravenous infusion of endotoxin to healthy volunteers caused a decrease in plasma ADAMTS13 : AC together with the appearance of UL-VWFM [77]. In patients with alcoholic hepatitis, especially in severe cases complicated by LC, ADAMTS13 : AC concomitantly decreased, and VWF : Ag progressively increased with increasing concentrations of these cytokines from normal range to over 100 pg/mL [25]. Plasma endotoxin concentration inversely correlated with ADAMTS13 activity and was higher in patients with UL-VWFM than that those without [25]. From these results as well as our own, marked cytokinemia and/or enhanced endotoxemia may be closely related to decreased ADAMTS13 : AC and the appearance of UL-VWFM [25]. It will be necessary to clarify what types of inhibitor may be involved in association with inflammatory cytokines and endotoxin.

## 7. Typical TTP in Patients with Liver Diseases

We previously encountered a patient with HCV-related LC who was compromised by fatal TTP [35]. This case showed advanced LC and rigid ascites. As reported in the literature, since 1979, there have been 13 patients with liver diseases who developed TTP [35, 79–90]. Five of them were treated with IFN therapy, but the remaining 8 were not. Three of them showed evidence of autoimmune hepatitis, one of which was complicated by systemic lupus erythematosus (SLE). The remaining 4 patients had HCV-related LC, hepatitis B virus- (HBV-) related LC, alcoholic LC, or haemochromatosis. IFN may be able to induce autoimmune reactions, resulting in the generation of autoantibodies against ADAMTS13, although this phenomenon has yet to be confirmed. On the other hand, irrespective of IFN therapy, HCV infection and/or advanced LC itself may contribute to the development of TTP. 

 There is general consensus that the overall prevalence of serum non-organ-specific autoantibodies is significantly higher in patients with HCV (about one third of all cases) than that in both healthy subjects and patients with HBV [91–93], but not alcoholic liver injury. In addition, HCV infection was confirmed in five of 10 patients (50%) who developed thrombotic microangiopathy (TMA) after living-donor liver transplantation [94]. In our study, the etiology of our five end-stage LC patients with IgG-type autoantibodies was HCV in 2, HBV in 1, PBC in 1, and cryptogenic in 1, but none of the patients displayed alcohol-abuse-related cirrhosis [30]. Nevertheless, the diagnosis of TTP may be hampered by clinical features accompanying hepatic failure similar to the pentad of typical TTP (fever, thrombocytopenia, renal failure, fluctuating neurological signs, and microangiopathic hemolytic anemia) [11, 12]. 

## 8. Possible Therapeutic Approaches of ADAMTS13 Supplementation for Patients with Decompensated LC

Fresh frozen plasma (FFP) infusion is commonly used to correct the prolonged prothrombin time in patients with advanced chronic liver disease, but exact indication for its use has not been clearly defined [95]. The aim of FFP infusions is usually either to improve the coagulopathy before invasive procedures or to control ongoing bleeding from various sites in patients with vitamin K-unresponsiveness prolonged prothrombin time. The mean prothrombin time was improved by the infusion of 2–6 units of FFP, but only 12.5% of the retrospective study group and 10% of the prospective study groups showed reversal of their coagulopathy, and higher volume (6 or more units) may be more effective but rarely is employed [96]. However, attention should be directed to complications including the risk of infection, allergic reaction, and acute volume expansion leading to heart failure or pulmonary edema [95, 96].

 With regard to FFP infusion as a unique source of ADAMTS13, we clearly showed that preexisting UL-VWFMs in the plasma of USS patients began to diminish within 1 hour and completely diminished 24 hours after ADAMTS13 was replenished with infusions of FFP [97]. Retrospectively, these results indicated that exogenous ADAMTS13 could efficiently cleave both UL-VWFMs that preexisted in the circulation and the newly produced molecules at the ECs surface. Advanced LC is known to be a predisposing state toward platelet microthrombi formation, even in the absence of clinically overt thrombi [30]. In our study, UL-VWFM-positive patients showed the lowest ADAMTS13 : AC and the highest values of serum creatinine, blood urea nitrogen, and blood ammonia, and the VWFM patterns appeared to shift from degraded to normal VWFM and finally to UL-VWFM as functional liver capacity and renal function deteriorated (Table 1). From these results, it may be reasonable to assume that advanced LC patients with severe to moderate deficiency of ADAMTS13 : AC (<3% to ~25% of normal control) could be candidates for FFP infusion as a source of ADAMTS13. It is necessary to evaluate the effectiveness of FFP administration to patients with ADAMTS13 : AC levels from 25% to 50%. 

Alternatively, our recent study demonstrated that plasma ADAMTS13 : AC is reduced in VOD patients after stem cell transplantation (SCT) (12–32% of normal) compared to non-VOD patients (57–78% of normal), even before any conditioning regimen and throughout SCT, and that the activity might thus be a predictor for the development of hepatic VOD [22]. A multicenter, prospective, randomized controlled study revealed that prophylactic FFP infusion may be instrumental in preventing the development of hepatic VOD after SCT [23]. The imbalance caused by decreased ADAMTS13 : AC versus increased production of VWF : Ag before and during the early stage after SCT would contribute to a microcirculatory disturbance that could ultimately lead to VOD [23]. The supplementation of ADAMTS13 by prophylactic FFP infusion may suppress the increase in VWF : AG that is extensively released from damaged SEC. Furthermore, we first reported in 2006 that a significant reduction of ADAMTS13 : AC with a concomitant appearance of UL-VWFM was consistently observed in patient plasma soon after liver transplantation [31]. These changes were closely related to liver-graft dysfunction, ischemia-reperfusion injury, and acute rejection. The ADAMTS13 : AC often decreased to less than 10% of normal controls, concurrent with severe thrombocytopenia. The organ dysfunction appeared to be restricted to the liver graft, indicating that a decrease of plasma ADAMTS13 : AC coupled with the appearance of UL-VWFM was attributed to a mechanism of “local TTP” within the liver graft [21, 31]. It is, therefore, extremely important to monitor plasma ADAMTS13 : AC in the treatment of thrombocytopenia associated with allograft dysfunction after liver transplantation. This is because the infusions of platelet concentrate under conditions of an imbalance of decreased ADAMTS13 : AC to enhanced UL-VWFM production might further exacerbate the formation of platelet aggregates mediated by uncleaved UL-VWFM, leading to graft failure via the “local TTP” mechanism [21, 31]. FFP infusion as ADAMTS13 replacement therapy may improve both liver dysfunction and thrombocytopenia in liver transplant patients. From this point of view, we are particularly interested in conducting clinical trials with recombinant ADAMTS13 preparations not only in patients with advanced LC but also in patients with VOD and liver transplantations. 

## 9. Conclusion and Future Perspectives

The introduction of ADAMTS13 to the field of hepatology not only enabled us to confirm the diagnosis of TTP early but also provided novel insight into the pathophysiology of liver diseases. Some diseases were shown to be TTP itself, but others did not show any apparent clinical features of TTP, even in the presence of extremely decreased ADAMTS13 : AC and increased UL-VWFM corresponding to TTP. Such TTP-like states, but without disseminated intravascular coagulation, might be “subclinical TTP” as seen in advanced liver cirrhotics [30] and SAH patients [24–27] or “local TTP” as shown in patients with hepatic VOD after SCT [22, 23] and patients with adverse events after living-donor liver transplantation [31, 32]. Essentially, one would be unable to detect such TTP-like phenomena without the determination of ADAMTS13 : AC, because the interaction of ADAMTS13 and UL-VWFM is the initial step in hemostasis, and their abnormalities do occur in the absence of apparent imbalance in other hemostatic factors and/or irrespective of the presence or absence of abnormal conventional hemostatic factors. The origin of VWF, the substrate of ADAMS13, indeed may be transformed hepatic sinusoidal and/or extrahepatic endothelial cells, but not hepatocytes. The procoagulant and anticoagulant proteins synthesized in hepatocytes decrease as liver disease progresses, whereas VWF markedly increases. Under such circumstances, ADAMTS13 deficiency may lead to a microcirculatory disturbance not only in the liver, but also in the systemic circulation. The determination of ADAMTS13 and its related parameters thus will be quite useful for improved understanding of the pathophysiology and for providing appropriate treatments especially in severe liver disease patients. It will be necessary to measure ADAMTS13 : AC when patients with unexplained thrombocytopenia are encountered in the course of liver disease. When “subclinical or local TTP” status would be confirmed, FFP infusion as ADAMTS13 replacement therapy may improve both liver dysfunction and thrombocytopenia. Further investigation will be necessary to define candidates for ADAMTS13 supplementation therapy and to evaluate its potential therapeutic efficacy in advanced LC patients.

## Figures and Tables

**Table 1:** Comparison of clinical parameters among cirrhotic patients according to VWF multimer patterns.

Variables	VWF multimer pattrens			a versus b	a versus c	b versus c
	Degraded^a^ (*n* = 15)	Normal^b^ (*n* = 26)	Unusually large^c^ (*n* = 8)			
ADAMTS13 : AC (%) (ELISA)	47 ± 24	44 ± 13	26 ± 14	n.s.	*P* < .05	*P* < .01
VWF : RCo (%)	110 ± 92	196 ± 134	216 ± 110	*P* < .05	*P* < .05	n.s.
Child-Pugh score	8.6 ± 2.5	10.9 ± 2.1	12.4 ± 1.7	*P* < .01	*P* < .005	n.s.
Serum albumin (g/dL)	3.07 ± 0.54	2.85 ± 0.54	2.59 ± 0.25	n.s.	*P* < .05	n.s.
Cholinesterase (IU/L)	126 ± 62	78 ± 64	60 ± 36	*P* < .05	*P* < .02	n.s.
Total cholesterol (mg/dL)	142 ± 51	93 ± 45	88 ± 40	*P* < .01	*P* < .03	n.s.
Hemoglobin (g/dL)	11.0 ± 1.7	9.3 ± 2.0	8.9 ± 1.7	*P* < .02	*P* < .02	n.s.
Serum creatinine (mg/dL)	1.06 ± 0.72	1.11 ± 0.79	2.43 ± 2.16	n.s.	*P* < .05	*P* < .03
Blood urea nitrogen (mg/dL)	22 ± 17	30 ± 21	74 ± 62	n.s.	*P* < .01	*P* < .01
Blood ammonia (*μ*g/dL)	87 ± 50	100 ± 39	144 ± 53	n.s.	*P* < .05	*P* < .05

VWF: von Willebrand factor; ADAMTS13 : AC : ADAMTS13 activity; ELISA : enzyme-linked immunosorbent assay; VWF : RCo: VWF ristocetin cofactor activity; n.s.: not significant. (Partially modified from Uemura et al., [30]).
